# Enhancing algal growth and nutrient recovery from anaerobic digestion piggery effluent by an integrated pretreatment strategy of ammonia stripping and flocculation

**DOI:** 10.3389/fbioe.2023.1219103

**Published:** 2023-06-29

**Authors:** Jun Qian, Chengyu Xu, Hanwu Song, Wenguang Zhou, Tatsuki Toda, Hongwu Li, Yoshiki Takayama, Mutsumi Sekine, Shinichi Koga, Jun Li, Jin Liu

**Affiliations:** ^1^ Key Laboratory of Poyang Lake Environment and Resource Utilization, Ministry of Education, and School of Resources and Environment, Nanchang University, Nanchang, China; ^2^ Faculty of Science and Engineering, Soka University, Tokyo, Japan

**Keywords:** anaerobic digestion piggery effluent, ammonia stripping, chemical flocculation, microalgae cultivation, nutrient recovery

## Abstract

Anaerobic digestion piggery effluent (ADPE) with a quite high ammonium (NH_4_^+^) concentration and turbidity (dark brown color) generally requires high dilution before microalgae cultivation, owing to its NH_4_^+^ toxicity and color inhibition to algal growth. An integrated pretreatment strategy of ammonia stripping and chemical flocculation may be a more practical pretreatment procedure for enhancing algae yield and nutrient recovery from anaerobic digestion piggery effluent. In this study, we determined the optimum pretreatment strategy of anaerobic digestion piggery effluent for subsequent microalgae cultivation and nutrient recovery. The results showed that the integrated anaerobic digestion piggery effluent pretreatment strategy of high-temperature ammonia stripping and chemical flocculation at a mixed dosage of 2 g L^−1^ polyaluminum chloride (PAC) and 40 mg L^−1^ cationic polyacrylamide (C-PAM), and 50 mg L^−1^ ammonium nitrogen (NH_4_^+^-N) enrichment provided maximum algal yield (optical density = 1.8) and nutrient removal (95.2%, 98.7%, 99.3%, and 78.5% for the removal efficiencies of total nitrogen, NH_4_^+^-N, total phosphorus, and chemical oxygen demand, respectively) from anaerobic digestion piggery effluent. The integrated pretreatment strategy is expected to become a more practical pretreatment procedure for enhancing algae yield and nutrient recovery from anaerobic digestion piggery effluent.

## 1 Introduction

Large-scale piggery wastewater is commonly treated by an anaerobic digestion (AD) process, which results in the intermittent release of AD piggery effluent (ADPE), specifically comprising high levels of nitrogen, phosphorus, and organic matter (Qian et al., 2022b). If ADPE is directly discharged into natural water bodies, it will lead to serious environmental pollution and ecological risk (Zhao et al., 2022). Microalgae-based wastewater treatment is one of the most promising technologies for advanced AD effluent (ADE) treatment and nutrient recovery (Qian et al., 2022c), owing to fast algal growth, high photosynthetic efficiency, and excellent nutrient uptake. Simultaneously, the harvested algal biomass can be developed for high-value products such as animal feed, biogas, biofuel, and valuable chemicals (Qian et al., 2022a; Chen et al., 2022). However, a quite high ammonium (NH_4_^+^) concentration and turbidity (dark brown color) are the current barriers to adopting algal cultivation for ADPE remediation because either of the two factors can inhibit algal growth. Algal cells during photosynthesis can result in a high pH level of ADPE, which can easily shift the chemical equilibrium from NH_4_^+^ to free ammonia (NH_3_) (Ayre et al., 2017). NH_3_ is toxic to most algal strains by diffusing through the cell membrane and accumulating in the algal cytoplasm because of its uncoupling effects on photosynthesis in chloroplasts (Crofts, 1966; Uggetti et al., 2014). In addition, the dark brown color of ADPE easily obstructs the passage of sunlight from water bodies, thus greatly inhibiting algal photosynthesis and growth (Liu et al., 2015). Therefore, ADPE should be properly pretreated to reduce NH_3_ toxicity and color inhibition for subsequent microalgae cultivation.

In most previous studies, high-dilution pretreatment of ADPE was the most convenient method to reduce NH_4_^+^ toxicity and color inhibition prior to microalgae cultivation. For example, *Chlorella vulgaris* attained the maximum biomass yield in 50-fold diluted ADPE at an NH_4_^+^-N concentration of 100 mg L^−1^ and chromaticity of 170, but its growth was inhibited in 10- and 20-fold diluted ADPE (Kwon et al., 2020). This might be because the NH_4_^+^-N concentration of 110 mg L^−1^ was critical for NH_4_^+^ tolerance in green algae, including *Chlorella* spp. (Collos and Harrison, 2014). Indeed, the high-dilution pretreatment cannot be widely applied in large-scale microalgae-based wastewater treatment because it needs a huge volume of freshwater. Ammonia stripping is a reliable pretreatment process to maintain the NH_4_^+^ level of ADPE below the NH_4_^+^ tolerance of microalgae, but the dark color of ADPE still inhibits the growth of microalgae (Li et al., 2019). Chemical flocculation can rapidly remove the color with only a small amount of flocculant, and the process is stable and controllable (El Bied et al., 2021). For example, in the study conducted by Depraetere et al. (2013), 0.2 mM ferric chloride and 200 mg L^−1^ cationic starch could remove chromaticities of 76% and 73% in piggery wastewater, respectively. Therefore, an integrated pretreatment strategy of ammonia stripping and chemical flocculation may be a feasible procedure to eliminate NH_4_ toxicity and color inhibition from ADPE for subsequent microalgae cultivation.

This study was conducted to solve the technical problems encountered in ADPE pretreatment for subsequent microalgae cultivation. In this study, the integrated ADPE pretreatment strategy of ammonia stripping and chemical flocculation, and subsequent microalgae cultivation was conducted to optimize the most suitable pretreatment condition for microalgae cultivation. Then, the optimal pretreated ADPE was supplemented with each of the different NH_4_^+^-N concentrations and used for microalgae cultivation to further enhance algal yield and nutrient removal from ADPE. The integrated pretreatment strategy is expected to become a more practical pretreatment procedure for enhancing algal yield and nutrient recovery from ADPE.

## 2 Materials and methods

### 2.1 ADPE preparation and flocculants

ADPE was obtained from an anaerobic digester of the Zhenghe Biogas Plant in Xinyu City, Jiangxi Province, China. The plant collected piggery manure to generate biogas and ADPE through a continuous stirred tank reactor (CSTR). All the collected ADPEs were filtered through a solid–liquid centrifugal separator (LLW800, KAIDI, Suzhou, China) to remove large particles and stored at 4°C before use. Polyaluminum chloride (PAC) and cation polyacrylamide (C-PAM) as flocculants were purchased from Gongyi Xinqi Polymer Co., Ltd., China.

### 2.2 High-temperature ammonia stripping device

The high-temperature ammonia stripping device mainly consisted of a stripping tower, circulating pump, water tank, air-blower, heating unit, and electronic control unit (Supplementary Figure S1). The stripping tower was made of double-layer stainless steel with an inner diameter of 154 mm, a height of 1,500 mm, and a working volume of 10 L. A porous material was filled inside the stripping tower to adsorb NH_3_. ADPE was pumped from the water tank along with the pipeline to the top of the inner layer of the stripping tower using the circulating pump at a constant flow rate and then sprayed downward using a nozzle to fully contact the porous material. The outer layer of the stripping tower was filled with 70°C hot water, which was supplied by a heating water tank. Compressed air from the air-blower was injected into the bottom of the stripping tower through a porous diffuser, and the airflow was controlled by a flowmeter. ADPE in the stripping tower was circulated from the bottom back to the water tank, which was equipped with a blender to homogeneously mix ADPE. Ammonia stripped from the top of the stripping tower was collected and absorbed by 10% (v/v) sulfuric acid (H_2_SO_4_) solution to recover ammonium sulfate, which was used as supplementation of the culture medium for subsequent microalgae cultivation.

### 2.3 Algal strain and pre-culture conditions

One algal strain used for ADPE remediation was isolated from a local water body surrounding the Nanchang Maiyuan Landfill Plant and named Nanchang University (NCU)-7, which was identified as *Chlorella sorokiniana*, based on our previous methods (Li et al., 2022). *C. sorokiniana* has been widely applied in wastewater remediation because it can achieve high biomass production and nutrient removal in mixotrophic cultivation (León-Vaz et al., 2019; Qian et al., 2020; Qian et al., 2021).

Algal cells were maintained with a 100-mL TAP medium (Supplementary Table S1) in a 250-mL Erlenmeyer flask. They were pre-cultured at 28°C ± 1°C with a continuous light intensity of 3,000 lux and set on an orbital shaker (TS-2102GZ, Tensuc, Shanghai, China) at a rotation speed of 120 rpm.

### 2.4 Cultivation of microalgae

Algal cells in the TAP medium were centrifuged at 6,000 rpm for 5 min to remove the supernatant, and the residual algal pellets were washed twice and resuspended in distilled water to prevent the influence of the nutrients from the medium. Subsequently, the algal suspension was inoculated into 200 mL culture medium (appropriately treated ADPE) at an initial optical density (OD) of ca. 0.5 in a 500-mL flask and cultivated at the same temperature, light, and rotation speed conditions as those in the algal pre-culture.

### 2.5 Experimental design

#### 2.5.1 ADPE pretreatment by high-temperature ammonia stripping

A total volume of 40-L ADPE was first pretreated using the high-temperature ammonia stripping device to reduce the ammonium concentration. The ADPE was circulated at a flow rate of 500 mL min^−1^ and three cycles with a total stripping period of 4 h. The airflow rate was set to 15 m^3^ h^−1^. After ammonia stripping, the pretreated ADPE was collected for further experiments.

#### 2.5.2 Flocculation pretreatment for microalgae cultivation

The pretreated ADPE by ammonia stripping was used in the flocculation experiment. The gradient concentrations of PAC (2, 4, 6, and 8 g L^−1^) and C-PAM (40, 80, and 120 mg L^−1^) were fully mixed in ADPE for flocculation pretreatment, and the mixed concentration in each treatment is listed in Table 1. Subsequently, the flocculation sediment in each treatment was removed by centrifugation at 2,000 rpm for 2 min to obtain the supernatant, which was used as the culture medium for microalgae cultivation to determine the optimum pretreatment for enhancing algal yield. Simultaneously, ADPE with ammonia stripping pretreatment but without flocculation was used as a control.

**TABLE 1:** Chromaticity, pH, and the concentrations of total nitrogen (TN), ammonium nitrogen (NH_4_^+^-N), total phosphorus (TP), and chemical oxygen demand (COD) in untreated ADPE; ADPE pretreated by high-temperature ammonia stripping; and ADPE pretreated by the ammonia stripping combined with different chemical flocculant dosages (the different mixed dosages of polyaluminum chloride (PAC) and cationic polyacrylamide (C-PAM)). Data are reported as the mean ± standard deviation (*n* = 3).

Treatment	PAC		C-PAM	TN	NH_4_^+^-N	TP	COD	Chromaticity	pH
	g L^−1^		mg L^−1^	mg L^−1^	mg L^−1^	mg L^−1^	mg L^−1^	PCU	
Non-treatment*	0		0	1,357.9 ± 4.6	1,250.5 ± 22.6	71.2 ± 1.0	5,757.8 ± 122.2	10,500 ± 72	8.8 ± 0.1
+S**	0		0	205.6 ± 3.2	120.9 ± 2.2	62.9 ± 0.4	4,646.7 ± 100.0	8,920 ± 50	9.2 ± 0.1
+S + C/M(2/40)***	2		40	136.5 ± 2.7	71.7 ± 1.2	18.8 ± 0.5	2,356.7 ± 61.1	3,915 ± 49	7.6 ± 0.1
+S + C/M(2/80)	2		80	130.5 ± 2.0	70.9 ± 0.4	17.8 ± 0.4	2,245.6 ± 61.1	3,175 ± 31	7.4 ± 0.3
+S + C/M(2/120)	2		120	129.0 ± 0.7	67.7 ± 0.4	16.6 ± 0.1	2,206.7 ± 100.0	2,955 ± 77	7.5 ± 0.3
+S + C/M(4/40)	4		40	94.3 ± 0.9	61.9 ± 1.0	5.3 ± 0.1	1,434.4 ± 72.2	1,540 ± 1	7.3 ± 0.1
+S + C/M(4/80)	4		80	85.7 ± 0.6	55.3 ± 0.4	3.3 ± 0.2	1,195.6 ± 77.7	980 ± 5	7.3 ± 0.0
+S + C/M(4/120)	4		120	80.0 ± 0.6	56.5 ± 0.8	3.2 ± 0.0	1,106.7 ± 22.2	915 ± 7	7.3 ± 0.1
+S + C/M(6/40)	6		40	71.5 ± 0.5	45.4 ± 0.6	2.8 ± 0.3	1,084.4 ± 22.2	882 ± 1	7.2 ± 0.1
+S + C/M(6/80)	6		80	68.7 ± 0.5	43.4 ± 2.2	2.7 ± 0.1	1,062.2 ± 66.7	835 ± 2	7.4 ± 0.1
+S + C/M(6/120)	6		120	63.7 ± 0.5	42.4 ± 3.2	2.0 ± 0.5	884.4 ± 11.1	629 ± 1	7.4 ± 0.1
+S + C/M(8/40)	8		40	52.6 ± 1.1	38.0 ± 0.8	0.8 ± 0.2	717.8 ± 5.6	153 ± 1	7.3 ± 0.1
+S + C/M(8/80)	8		80	53.5 ± 1.9	34.2 ± 1.0	0.3 ± 0.1	706.7 ± 5.6	155 ± 2	7.3 ± 0.1
+S + C/M(8/120)	8		120	53.1 ± 0.2	40.2 ± 1.0	0.2 ± 0.0	695.6 ± 11.1	143 ± 1	7.5 ± 0.2

*ADPE without high-temperature ammonia stripping and chemical flocculation.

**ADPE pretreated by high-temperature ammonia stripping.

***ADPE pretreated by ammonia stripping combined with chemical flocculation at a mixed dosage of 2 g L^−1^ PAC and 40 mg L^−1^ C-PAM.

#### 2.5.3 Microalgae cultivation in the optimum pretreated ADPE enriched with different NH_4_^+^-N concentrations

To further enhance the algal yield and nutrient removal from ADPE, the algal cells were cultured in the optimum pretreated ADPE enriched with NH_4_^+^-N concentrations of 50, 100, and 200 mg L^−1^. The optimum pretreated ADPE without NH_4_^+^-N enrichment was used as a control. These NH_4_^+^-N concentrations were provided by the recovered ammonium sulfate from the ammonia stripping device.

### 2.6 Analytical methods

#### 2.6.1 Algal growth and water quality analysis

OD as algal yield was measured at 680 nm using a spectrophotometer (DR 6000, Hach, Loveland, United States) to determine the growth of microalgae. Chlorophyll *a* in algal cells was measured based on a previous study (Song et al., 2020).

pH and chromaticity (unit: PCU) were measured using a portable pH meter (PB-10, Sartorius, Beijing, China) and colorimeter (SD9011B, XINRUI, Shanghai, China), respectively. To determine the water quality characteristics, the collected sample was filtered through a 0.45-µm pore-size syringe filter (PES, Jinteng, Tianjin, China) to remove suspended materials. The filtrate was analyzed using a Hach kit (DR 6000 Hach, Loveland, United States) to determine the concentrations of the chemical oxygen demand (COD), total nitrogen (TN), NH_4_^+^-N, and total phosphorus (TP). The removal efficiencies (*R*, %) of nutrients (COD, TN, NH_4_^+^-N, and TP) were calculated using the following equation:

$$R=\left(C_{0i}–C_{fi}\right)/\;C_{0i}\times \left(100\right),$$ (1)

where *C*_0*i*_ and *C*_f*i*_ represent the concentrations (mg L^−1^) of nutrient *i* on the initial and final days of the experimental period, respectively.

#### 2.6.2 Three-dimensional fluorescence excitation–emission matrix (3D-EEM) analysis

The EEM spectra of the ADPE samples were determined by fluorescence spectroscopy (F97pro, Lengguang Tech, Shanghai, China) with a scanning emission (Em) wavelength from 300 to 600 nm (1-nm increment) by increasing the excitation (Ex) wavelength from 300 to 500 nm (5-nm increments).

## 3 Results and discussion

### 3.1 ADPE pretreated by the integrated strategy and corresponding EEM analysis

In untreated ADPE, chromaticity, TN, NH_4_^+^-N, TP, and COD concentrations were 10,500 PCU, 1,357.9, 1,250.5, 71.2, and 5,757.8 mg L^−1^, respectively, while they decreased to 8,920 PCU, 205.6, 120.9, 62.9, and 4,646.7 mg L^−1^ after high-temperature ammonia stripping pretreatment (+s), respectively (Table 1). The slight decrease in COD and chromaticity may be because the volatile organic compounds escape from ADPE through heat degradation and CH_4_ production (Meng et al., 2023)_._ The NH_4_^+^-N content sharply declined, but chromaticity was still quite high after ammonia stripping. However, after the integrated pretreatment of ammonia stripping combined with chemical flocculation, chromaticity, TN, NH_4_^+^-N, TP, and COD concentrations further reduced to 2,950–3,920 PCU, 129–137, 67–72, 16–19, and 2,200–2,360 mg L^−1^ in +S + C/M(2/40-120) treatments, respectively (e.g., +C/M(2/40) represented chemical flocculation at a mixed dosage of 2 g L^−1^ PAC and 40 mg L^−1^ C-PAM). With the increasing PAC supplementation amounts from 4 to 8 g L^−1^, they further declined to 910–1,550 PCU, 80–95, 55–62, 3.2–5.3, and 1,100–1,440 mg L^−1^ in +S + C/M(4/40-120) treatments; 620–890 PCU, 63–72, 42–46, 2.0–2.8, and 880–1,090 mg L^−1^ in +S + C/M(6/40-120) treatments; and 140–155 PCU, 52–54, 34–41, 0.2–0.8, and 690–720 mg L^−1^ in +S + C/M(8/40-120) treatments. These results suggested that the chemical flocculation of PAC and C-PAM addition could not only effectively reduce COD and chromaticity from ADPE but also remove nutrients including nitrogen and phosphorus. Compared to the C-PAM dosage, the increases in PAC dosage could more effectively remove TN, NH_4_^+^-N, TP, COD, and chromaticity from ADPE. Furthermore, the increases in PAC dosage had a greater removal effect on COD and chromaticity than TN, NH_4_^+^-N, and TP. These results were confirmed by the study conducted by Harif et al. (2023).

It could be found that the color removal effect of the flocculant on ADPE was not good enough when PAC dosage was low, the formed floc was small and suspended in ADPE, and the floc sedimentation performance was poor (Supplementary Figure S2). With the increases in PAC dosages, floc formation was accelerated in ADPE, the floc volume became larger, and the sedimentation performance of floc improved, corresponding to the enhancement of color removal. As known, the flocculation of PAC is mainly related to the charge neutralization process or the adsorption and precipitation of metal hydroxide. The deposition of hydroxyl radicals leads to the possibility of sweep flocculation, in which the impurity particles are involved in the growing sediment, thus being effectively removed (Duan and Gregory, 2003). The addition of PAC to wastewater would destroy the stability of colloidal materials and make small particles agglomerate into large settleable flocs (Wei et al., 2016).

**FIGURE 1:**
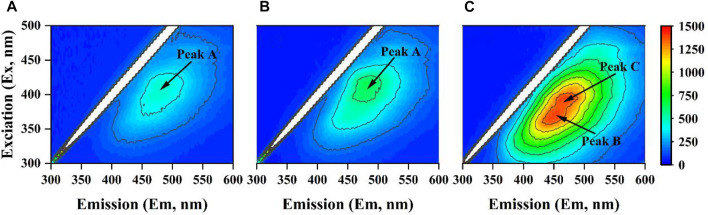
Three-dimensional excitation—emission matrix (EEM) spectra of untreated ADPE **(A)**, ADPE pretreated by high-temperature ammonia stripping **(B)**, and ADPE pretreated by high-temperature ammonia stripping and chemical flocculation **(C)** [where the mixed dosages of flocculants were 2 g L^−1^ polyaluminum chloride (PAC) and 40 mg L^−1^ cation polyacrylamide (C-PAM)].

3D-EEM spectroscopy was used to evaluate the structure characterizations of organic matter in ADPE before and after ammonia stripping combined with chemical flocculation pretreatments. Peak A was only identified from ADPE in non-treatment and +s treatment at Ex/Em 390–430/450–500 nm (Figures 1A, B), while peaks B and C were detected in +S + C/M(2/40) treatment at Ex/Em 350–380/430–460 nm and 380–410/460–490 nm, respectively (Figure 1C). These three peaks were all described as polycarboxylate-type humic acid (Wang and Zhang, 2010; Qian et al., 2023).

### 3.2 Microalgae cultivation in pretreated ADPEs by the integrated strategy

**FIGURE 2:**
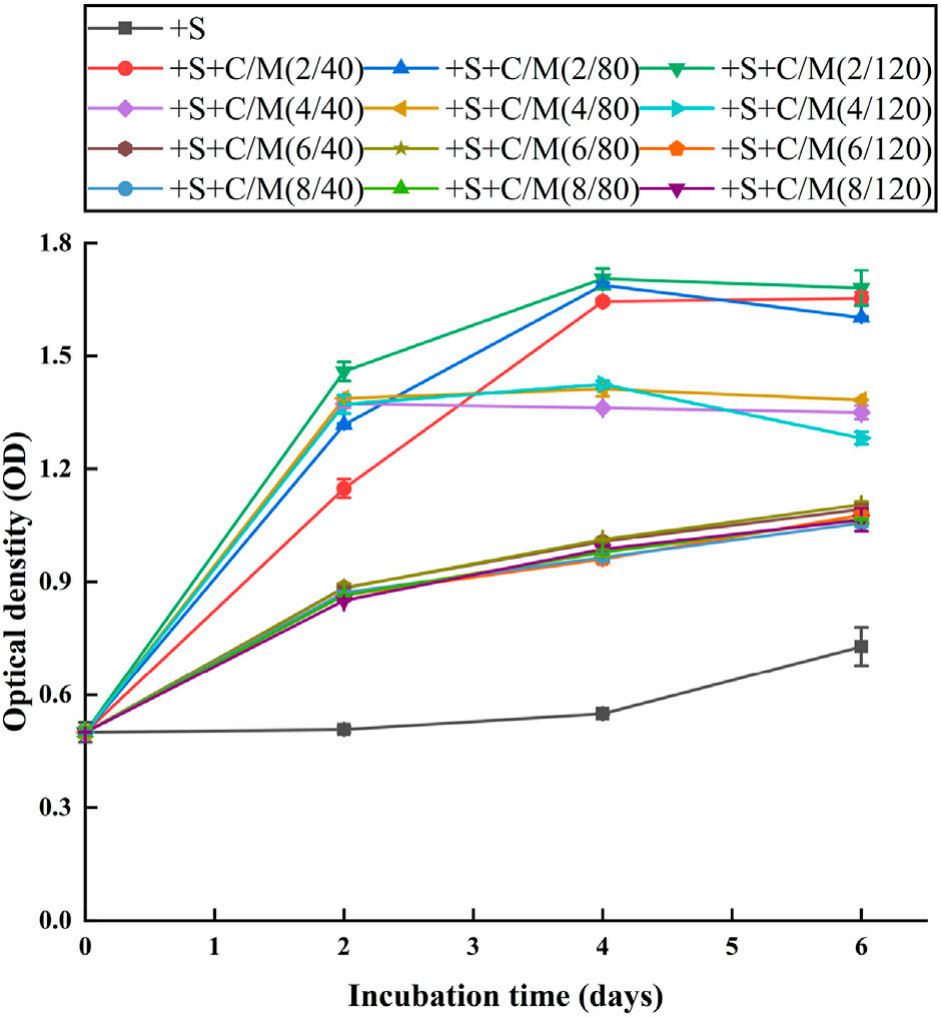
Growth curves of NCU-7 in different treatments (detailed information on each treatment is shown in Table 1). Error bars represent standard deviations (*n* = 3).

Algal growth was inhibited in +S treatment without flocculation (Figure 2) probably because the extremely high chromaticity (ca. 9,000 PCU) still existed in the pretreated ADPE. Very high chromaticity easily leads to the light reduction received by microalgae and inhibits the synthesis of various compounds in algal cells (Maltsev et al., 2021). ODs as algal yields were the highest in +S + C/M(2/40-120) treatment, being ca. 1.6, on the final day among the treatment, followed by ca. 1.3 in +S + C/M(4/40-120) treatment and ca. 1.1 in +S + C/M(6 and 8/40-120) treatment. This suggested that ADPEs in +S + C/M(2/40-120) treatment with a PAC supplementation of 2 g L^−1^ were more suitable media for microalgae cultivation, maybe due to a large amount of phosphorus existing in the media (Table 1) compared to the other treatment procedures. To reduce C-PAM dosage as much as possible for high cost efficiency, adding the mixed supplementation of 2 g L^−1^ PAC and 40 mg L^−1^ C-PAM to the ammonia stripping-pretreated ADPE (i.e., +S + C/M(2/40) treatment) was the most suitable pretreatment for enhancing algal yield from ADPE. Wang et al. (2020) also conducted a similar study, which reported that the COD, total Kjeldahl nitrogen (TKN), and TP contents in dairy manure wastewater were removed by 72.6%, 58.7%, and 43.0% in the optimal flocculation–centrifuge pretreatment, respectively, and their content in the two-fold diluted manure supernatant from this pretreatment was further reduced by 82.2%, 90.1%, and 83.4%, respectively, with a harvested algal yield of ca. 1.2 g L^−1^ during culturing *C. vulgaris* UTEX-2714. These results confirmed that the integrated pretreatment strategy of ammonia stripping and chemical flocculation was a reliable procedure to avoid NH_3_ toxicity and color inhibition from ADPE for subsequent microalgae cultivation.

### 3.3 Microalgae cultivation in pretreated ADPEs enriched with different NH_4_^+^-N concentrations

Based on the most suitable pretreatment, +S + C/M(2/40)-pretreated ADPEs were enriched with different NH_4_^+^-N concentrations and used as culture media for microalgae cultivation to further enhance algal yield and nutrient removal from ADPE. These NH_4_^+^-N concentrations were provided by recovered ammonium sulfate from the ammonia stripping device to achieve nitrogen resource recycling and utilization**.**

**FIGURE 3:**
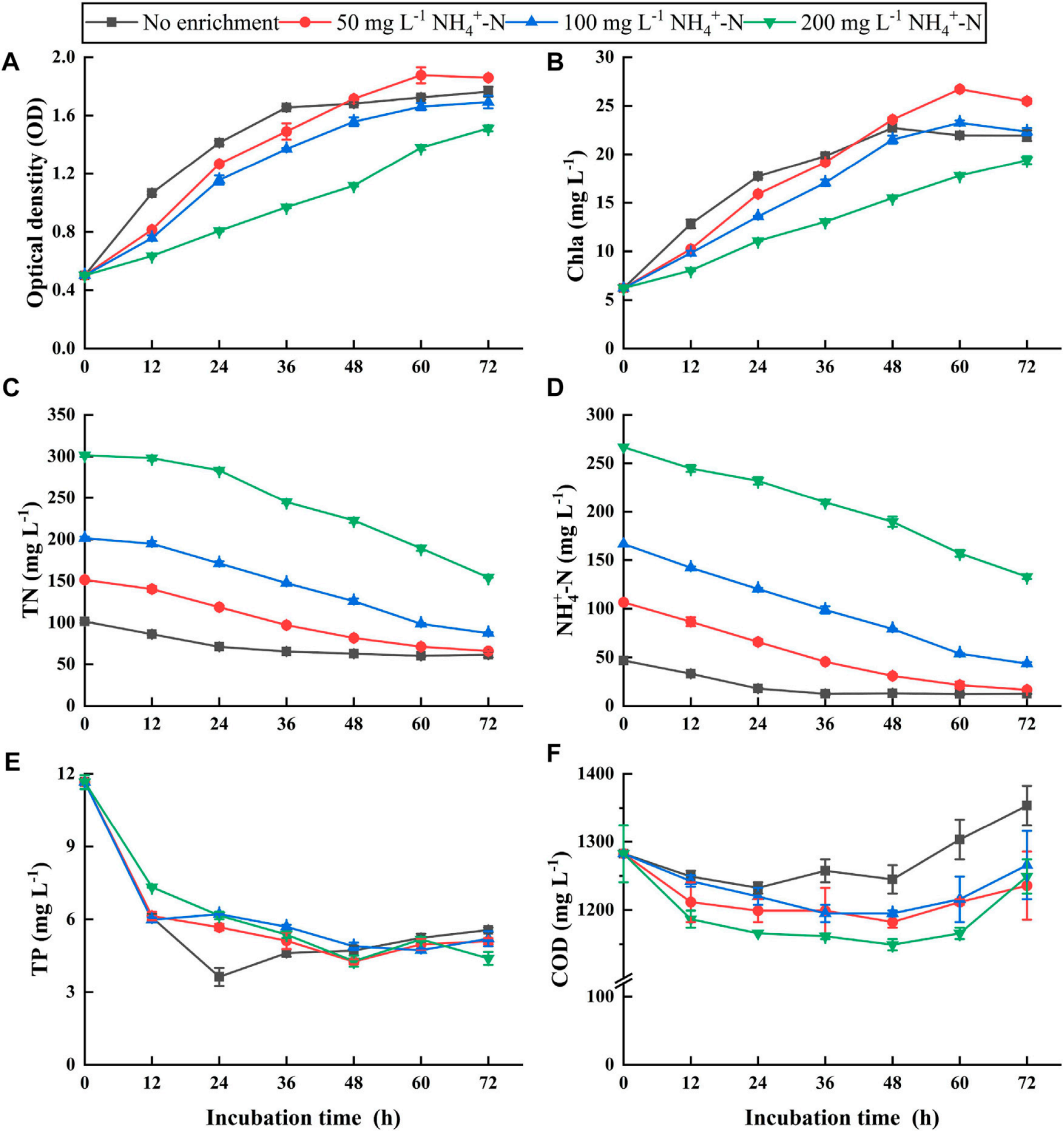
Algal growth curves **(A)**, chlorophyll a **(B)**, and the concentrations of total nitrogen (TN) **(C)**, ammonium nitrogen (NH_4_^+^-N) **(D)**, total phosphorus (TP) **(E)**, and chemical oxygen demand (COD) **(F)** in the optimum pretreated ADPE enriched with NH_4_^+^-N concentrations of 50, 100, and 200 mg L^−1^ (“the optimum pretreated ADPE” represents “ADPE was pretreated by the ammonia stripping combined with chemical flocculation at a mixed dosage of 2 g L^−1^ PAC and 40 mg L^−1^ C-PAM”). Error bars represent standard deviations (*n* = 3).

Growth curves of NCU-7 showed similar trajectories among all treatments, but the final algal yields were quite different. OD was ca. 1.8 in the 50-mg NH_4_^+^-N treatment, being the highest among the treatments, followed by 1.6 in no enrichment and 100-mg NH_4_^+^-N treatment (Figure 3A). However, the lowest algal yield (OD = 1.5) was obtained in the 200-mg NH_4_^+^-N treatment. The trends of algal yields among the treatments were similar to those of chlorophyll *a* (Figure 3B)*.* These results suggested that algal growth might be inhibited in 100- and 200-mg NH_4_^+^-N treatments compared to the 50-mg NH_4_^+^-N treatment. Therefore, the 50-mg NH_4_^+^-N enrichment was a suitable treatment for microalgae cultivation.

TN, NH_4_^+^-N, and TP concentrations in all treatments decreased with increasing incubation times, regardless of the initial concentrations. At the end of the experiment, 50-, 100-, and 200-mg NH_4_^+^-N treatments, and no enrichment removed 85.5, 114.1, 133.8, and 34.3 mg L^−1^ of TN concentrations (Figure 3C), respectively, in response to NH_4_^+^-N removal of 90.2, 123.1, 147.3, and 39.8 mg L^−1^, respectively (Figure 3D). It has been known that high pH levels above 8.0 favor NH_4_^+^ volatilization by converting into ammonia (Escudero et al., 2014). Therefore, this TN/NH_4_^+^-N removal might include partial volatilization losses during the experiment due to pH levels of 9.0–11.0 (data not shown). TP concentrations decreased to almost the same level in all treatments (Figure 3E). pH levels above 7.0 contributed to enhancing phosphorus removal from wastewater through phosphate precipitation with metals, such as calcium phosphate and/or struvite (Moutin et al., 1992; Le Corre et al., 2009). The level of algal yields in the 50-mg NH_4_^+^-N treatment was significantly higher than that in the 100- and 200-mg NH_4_^+^-N treatments despite a lower TN/NH_4_^+^-N removal amount in the 50-mg NH_4_^+^-N treatment and similar levels of TP removal amounts among the three treatments. This suggested that relatively high nitrogen and phosphorus removal was assimilated into the algal biomass in the 50-mg NH_4_^+^-N treatment. COD concentrations gradually decreased until 48 h but increased after that point (Figure 3F). The decrease was ascribed to algal heterotrophy, while the increase might be related to algal excretion (Zhuang et al., 2016). Based on the initial nutrient concentrations in raw ADPE without pretreatment, TN, NH_4_^+^-N, TP, and COD in the suitable 50-mg NH_4_^+^-N treatment with high algal yields achieved high removal efficiencies of 95.2%, 98.7%, 99.3%, and 78.5%, respectively.

## 4 Conclusion

The integrated ADPE pretreatment strategy of high-temperature ammonia stripping and chemical flocculation at a mixed dosage of 2 g L^−1^ PAC and 40 mg L^−1^ C-PAM, and 50 mg L^−1^ NH_4_^+^-N enrichment provided the maximum algal yield (OD = 1.8) and nutrient removal (95.2%, 98.7%, 99.3%, and 78.5% for TN, NH_4_^+^-N, TP, and COD removal efficiencies, respectively) from ADPE. The integrated pretreatment strategy could be a promising approach to algal biomass production and nutrient removal from ADPE.

## Data Availability

The original contributions presented in the study are included in the article/Supplementary Material; further inquiries can be directed to the corresponding authors.
