# Supplementary material for: Enhancing algal growth and nutrient recovery from anaerobic digestion piggery effluent by an integrated pretreatment strategy of ammonia stripping and flocculation
Source: Front Bioeng Biotechnol. 2023 Jun 29;11:1219103. doi: 10.3389/fbioe.2023.1219103 (PMC10339316; doi:10.3389/fbioe.2023.1219103)
Supplement: Supplementary file 1 [file DataSheet1.docx]

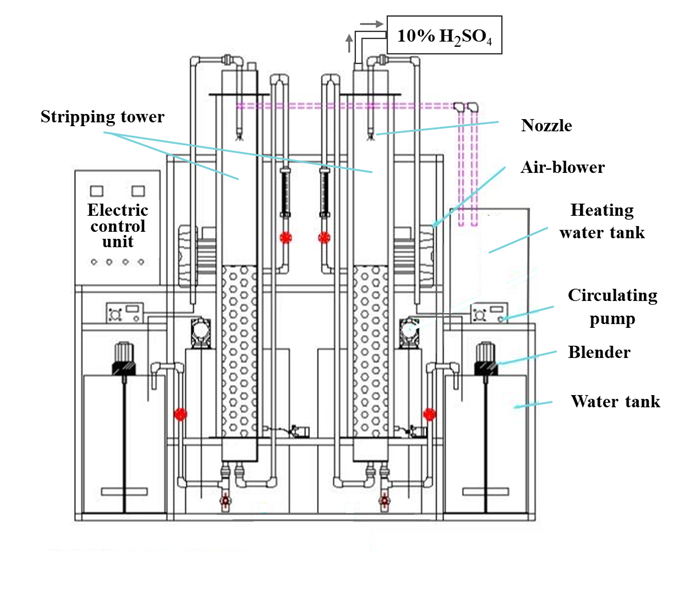


**Fig. S1.** Schematic of the high-temperature ammonia stripping device


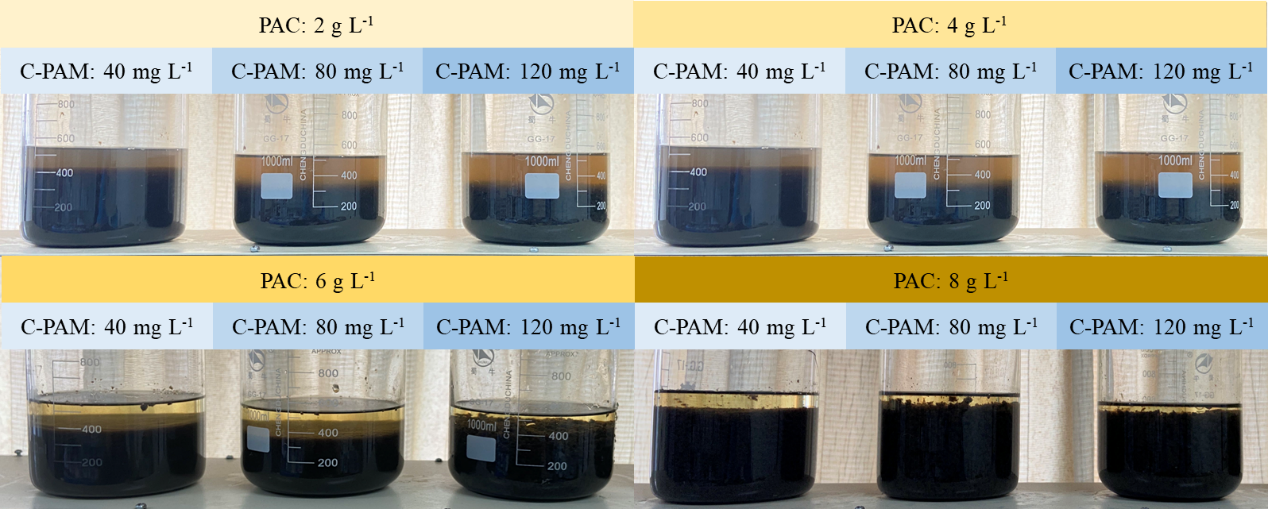


**Fig. S2.** Floc formation in high-temperature ammonia-striping pretreated ADPE under different mixed dosages of polyaluminum chloride (PAC) and cationic polyacrylamide (C-PAM)

**Table S1**

Chemical composition of tris-acetate-phosphate (TAP) medium with 1 mL L^-1^ of acetic acid.

| **Chemicals** | **Concentration**  **(mg L^-1^)** |
| --- | --- |
| Tris salt | 2420 |
| NH_4_Cl | 375 |
| CaCl_2_•2H_2_O | 50 |
| MgSO_4_•7H_2_O | 100 |
| K_2_HPO_4_ | 288 |
| KH_2_PO_4_ | 144 |
| Na_2_EDTA•2H_2_O | 50 |
| ZnSO_4_•7H_2_O | 22 |
| H_3_BO_3_ | 11.4 |
| MnCl_2_•4H_2_O | 5 |
| FeSO_4_•7H_2_O | 5 |
| CoCl_2_•6H_2_O | 1.6 |
| CuSO_4_•5H_2_O | 1.6 |
| (NH_4_)_6_Mo_7_O_24_•4H_2_O | 1.1 |
